# Exophthalmos as the initial presentation of metastatic prostate cancer

**DOI:** 10.1016/j.radcr.2024.09.060

**Published:** 2024-09-23

**Authors:** Matthijs Duijn, Marina C. Hovius, Gyorgio J. Seedo, Philippe C. Baars, Yves J.L. Bodar

**Affiliations:** a Department of Urology, Amsterdam University Medical Centers, University of Amsterdam, Amsterdam, The Netherlands; b Depatment of Urology, Amsterdam, The Netherlands; c Department of Neurology, Amsterdam, The Netherlands; d Department of Radiology, Amsterdam, The Netherlands

**Keywords:** Prostate cancer, Malignant tumor, Metastases, Orbital metastases, Hormonal therapy, PSMA PET/CT, ^18^F-PSMA-1007 tracer

## Abstract

Primary metastatic prostate cancer to the orbit is exceedingly rare. Benign lesions, including meningioma, have demonstrated PSMA expression and can be visualized using PSMA-based PET tracers. We report the findings of ^18^F-PSMA-1007 PET/CT in a 76-year-old man with progressive confusion and long-standing blindness of the left eye. PET/CT scan revealed increased uptake of PSMA in the orbital and temporal region, and other sites throughout the body. Histopathological examination after biopsy of the left orbit showed adenocarcinoma of the prostate. This case substantiates the diverse clinical and radiological presentations of metastatic prostate cancer and underscores the diagnostic significance of targeted biopsy.

## Introduction

Prostate cancer (PCa) is the second most prevalent malignancy in men worldwide [1]. The diagnosis of primary metastatic disease is not uncommon. Approximately 13% of all new cases exhibit locoregional lymph node involvement and 8% manifest distant metastasis [2]. The 5-year relative survival rate of metastatic prostate cancer is 32% [3]. Most common sites of distant metastasis are bone (84%), followed by distant lymph nodes (10.6%). Prevalent sites of bone metastasis related to PCa are typically the spine, pelvis and ribs [4]. In some cases, carcinomas metastasize to the eye and orbit [5]. However, primary metastatic PCa to the orbit has rarely been described in the literature as a primary presentation of the disease [[6], [7], [8]]. Symptoms may include exophthalmos, pain, decreased vision and periorbital swelling [5]. Benign lesions, including meningioma, can present similarly. Meningioma is the most common primary brain tumor, accounting for a one-third of all central nervous system tumors [9]. Meningiomas have demonstrated PSMA expression and can be visualized using PSMA-based positron emission tomography (PET) tracers [8,10]. This case highlights the necessity of excluding alternate diagnoses by integrating multiple imaging modalities and histopathological examination during the diagnostic process.

## Case report

A 76-year-old male patient presented at the emergency department exhibiting progressive confusion and long-standing blindness of the left eye. No other neurological symptoms were present. The patients’ medical history included type II diabetes mellitus and hypercholesterolemia. No relevant urological history or complains were documented. The patient adhered to regular medication, including insulin and simvastatin. Neurological examination revealed no abnormalities.

**Fig. 1:**
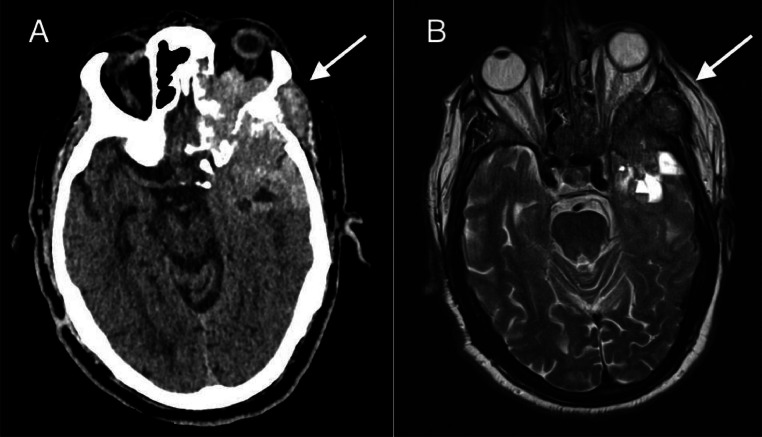
(A) Axial contrast-enhanced CT of the brain showed an irregular infiltrative hyperdense mass in the left temporal region (arrow), characterized by necrotic components and extensive osseous destruction. The mass exhibited orbital, sinusal and muscular extension. Exophthalmos on the left side, adjacent vasogenic edema with increased mass effect and some midline-shift were observed. (B) T2-weighted MRI image showed an hyperintense lesion left temporal with osseous extension (arrow).

Axial contrast-enhanced computed tomography (CT) of the brain showed an irregular infiltrative hyperdense mass in the left temporal region, characterized by necrotic components and extensive osseous destruction. The mass exhibited orbital, sinusal and muscular extension. Exophthalmos on the left side, adjacent vasogenic edema with increased mass effect and some midline-shift were observed (Fig. 1A). Subsequent T2-weighted magnetic resonance imaging (MRI) image showed an hyperintense lesion left temporal with osseous extension (Fig. 1B).

Serum prostate-specific antigen (PSA) levels, measured using the Roche assay, were 194 ng/mL. These findings raised suspicion for metastatic prostate cancer and prompted histopathological evaluation of the intraorbital lesion. After consent, a biopsy of the abnormality was conducted. Histological analysis confirmed the presence of adenocarcinoma of the prostate.

**Fig. 2:**
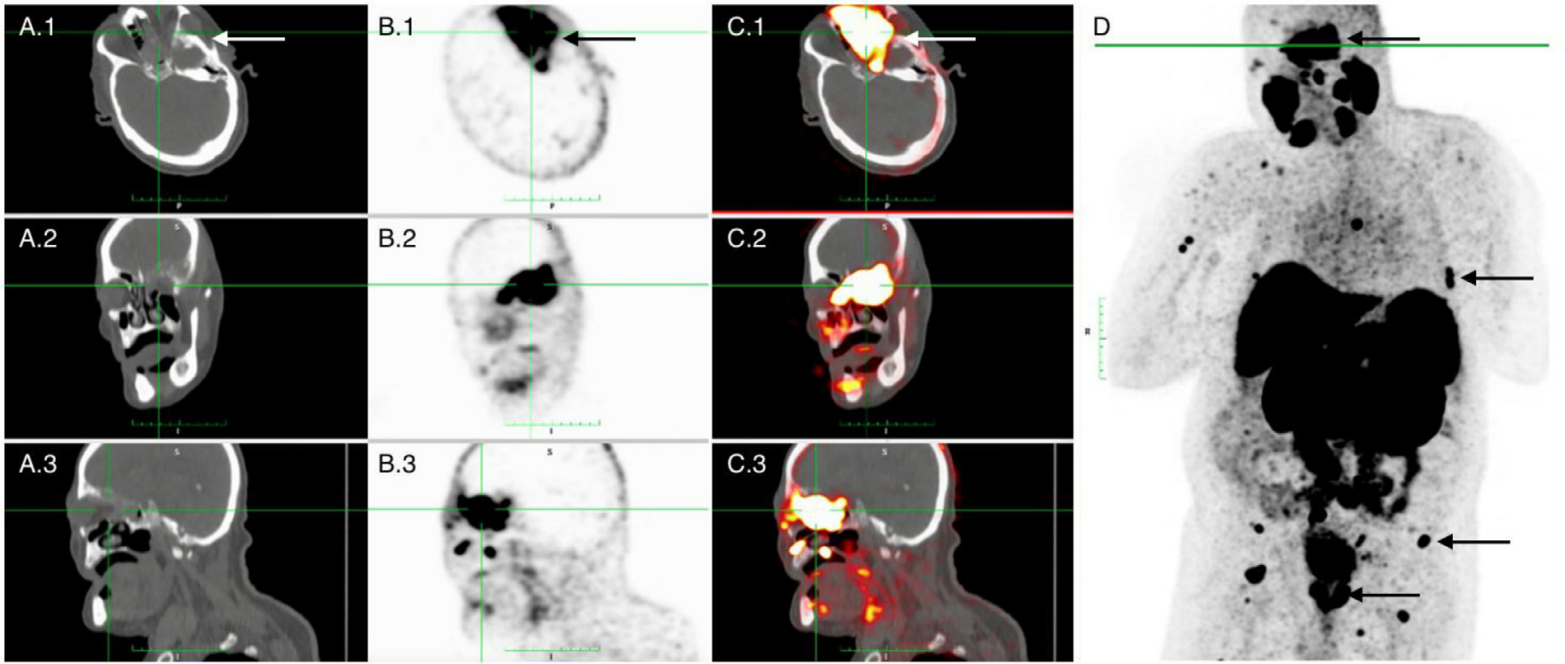
Axial, coronal and sagittal CT (A.1-A.3) and axial, coronal and sagittal PET images (B.1-B.3) revealed an intense PSMA focus in the left orbit with soft tissue involvement on CT (arrow). Fused PET/CT imaging by ^18^F-PSMA-1007 displayed the orbital lesion with intense tracer activity (arrow; C.1-C.3). Additionally, the maximum intensity projection (MIP) of ^18^F-PSMA-1007 PET/CT (D) showed the PSMA-avid orbital lesion (arrow 1), extensive bone metastases throughout the body (arrow 2), strong PSMA uptake in multiple (loco)regional lymph nodes (arrow 3), and intense tracer expression in both sides of the prostate with invasion of the left seminal vesicle (arrow 4).

^18^F-PSMA-1007 PET/CT was performed for additional staging. Axial, coronal and sagittal CT (Fig. 2A1-3) and axial, coronal and sagittal PET images (Fig. 2B1-3) revealed an intense PSMA focus in the left orbit with soft tissue involvement on CT. Fused PET/CT imaging by ^18^F-PSMA-1007 displayed the orbital lesion with intense tracer activity (Fig. 2C1-3). Additionally, the maximum intensity projection (MIP) of ^18^F-PSMA-1007 PET/CT (Fig. 2D) showed the PSMA-avid orbital lesion, extensive bone metastasis throughout the body, strong PSMA uptake in multiple (loco)regional lymph nodes, and intense tracer expression in both sides of the prostate with invasion of the left seminal vesicle. The current staging indicated miT3bN1M1b high volume adenocarcinoma of the prostate.

The patient initially commenced androgen deprivation therapy (ADT) with testosterone receptor antagonists for the course of 3 weeks (bicalutamide), followed by luteinizing hormone-releasing hormone (LHRH) agonists (Triptorelin). Consideration for local external orbital radiotherapy in conjunction with dexamethasone was proposed. Follow-up will be in 3 months with PSA and testosterone monitoring.

## Discussion

About 90 to 95% of prostate cancers are acinar adenocarcinomas that originate in the peripheral zone of the prostate gland [11]. The histological diagnosis is established by evaluating the absence of basal cells, disruption of normal glandular architecture, such as the disintegration of the epithelial-stromal basement membrane and the presence of nuclear atypia in luminal cells [12]. The aggressiveness of the adenocarcinoma is determined by the degree of histological differentiation, which is assessed using the Gleason Score or International Society of Urologic Pathology (ISUP) grading system [13]. PCa patients with higher Gleason or ISUP score are more likely to present with higher incidence of regional lymph nodes and distant metastasis [14].

Approximately 13% of all newly diagnosed PCa cases exhibit locoregional lymph node involvement, while 8% manifest distant metastasis [2]. The most common sites of bone metastasis in PCa are the spine, pelvis and ribs [4]. Pain is the predominant symptom, affecting 75% of patients with metastatic PCa who exhibit symptoms, while spinal cord compression occurs in up to 12% of cases and may be an initial indication of bone metastasis [15,16]. In rare instances, other uncommon bone parts of the body are affected. Primary metastasis of PCa to the orbit has been documented in a handful of cases [6−8]. In such cases, patients may exhibit a triad of symptoms consistent with spheno-orbital meningioma, including proptosis, visual impairment, and ocular paresis. Additionally, headache and periorbital swelling may also be observed [5,17].

Meningioma is the most common primary brain tumor, comprising approximately one-third of all central nervous system neoplasms [9]. MRI with godalinium-based contrast agents is the preferred modality for the evaluation of meningiomas. These tumors typically manifest as lobulated, extra-axial masses with well-circumscribed margins and often show homogenous enhancement with gadolinium contrast. On MRI, they usually exhibit signal intensity characteristics that are isointense to slightly hypointense relative to grey matter on T1-weighted sequences and isointense to slightly hyperintense relative to grey matter on T2-weighted sequences [18]. Previous studies have shown that meningiomas express PSMA-based PET tracers within their endothelial cells, leading to diagnostic confusion if the patient's clinical and radiological characteristics, such as MRI findings, are not carefully integrated [8,10].

The patient in our case presented with progressive cognitive impairment and long-standing blindness of the left eye. T2-weighted MRI imaging revealed a hyperintense lesion in the left temporal lobe with evidence of osseous involvement, raising suspicion for a more aggressive pathology (Fig. 1B). These radiological characteristics made meningioma an unlikely diagnosis. PSA levels of 194 ng/mL warranted additional staging through ^18^F-PSMA-1007 PET/CT and histopathological examination. Histological analysis confirmed adenocarcinoma of the prostate. ^18^F-PSMA-1007 PET/CT showed multiple (loco)regional lymph nodes and extensive skeletal metastasis, current staging miT3bN1M1b high volume disease. The patient was deemed unsuitable for chemotherapy with docetaxel and/or abiraterone with prednisolone and was started on ADT monotherapy.

Metastatic PCa demonstrates a wide spectrum of clinical manifestations and can occasionally present in a manner mimicking other pathological entities, such as meningioma. However, prostate metastases, particularly in advanced stages, are typically associated with more aggressive disease behavior. The signal intensity of metastatic lesions may vary depending on the degree of tissue necrosis or hemorrhage. This case highlights the variability in radiologic features among distinct pathologies and emphasizes the necessity of integrating supplementary imaging modalities and histopathological evaluation into the diagnostic process to accurately exclude differential diagnoses.

## Conclusion

This case underscores the importance of considering metastatic prostate cancer in the differential diagnosis of orbital masses, especially in elderly males presenting with atypical symptoms and elevated PSA levels. The integration of advanced imaging modalities like ^18^F-PSMA-1007 PET/CT and histopathological examination is critical in establishing an accurate diagnosis, guiding treatment decisions, and ultimately improving patient outcomes.

## Patient consent

I, as first and corresponding author, confirm that written informed consent for publication of this case report was obtained from the patient itself.
